# Decreased renal function increases the nighttime urine volume rate by carryover of salt excretion to the nighttime

**DOI:** 10.1038/s41598-021-90166-x

**Published:** 2021-05-19

**Authors:** Kentaro Takezawa, Sohei Kuribayashi, Koichi Okada, Yosuke Sekii, Yusuke Inagaki, Shinichiro Fukuhara, Hiroshi Kiuchi, Toyofumi Abe, Kazutoshi Fujita, Motohide Uemura, Ryoichi Imamura, Norio Nonomura

**Affiliations:** 1 Department of Urology, Osaka University Graduate School of Medicine, 2-2, Yamada-oka, Suita, Osaka 565-0871 Japan; 2 Department of Urology, Faculty of Medicine, Kindai University, Sayama, Osaka Japan

**Keywords:** Kidney diseases, Urological manifestations

## Abstract

To determine the pathophysiology of nocturnal polyuria associated with renal dysfunction, patients who underwent laparoscopic nephrectomy were prospectively studied. The diurnal variation in urine volume, osmolality, and salt excretion were measured on preoperative day 2 and postoperative day 7. The factors associated with an increase in the nighttime urine volume rate with decreased renal function were evaluated using multiple linear regression analysis. Forty-nine patients were included. The estimated glomerular filtration rate decreased from 73.3 ± 2.0 to 47.2 ± 1.6 mL/min/1.73 m^2^ (*P* < 0.01) and the nighttime urine volume rate increased from 40.6% ± 2.0% to 45.3% ± 1.5% (*P* = 0.04) with nephrectomy. The nighttime urine osmolality decreased from 273 ± 15 to 212 ± 10 mOsm/kg and the nighttime salt excretion rate increased from 38.7% ± 2.1% to 48.8% ± 1.7% (both *P* < 0.01) with nephrectomy. Multiple linear regression analysis showed that the increase in the nighttime urine volume rate was strongly affected by the increase in the nighttime salt excretion rate. A decrease in renal function causes an increase in the nighttime urine volume rate, mainly because of an increase in nighttime salt excretion.

Trial registration number: UMIN000036760 (University Hospital Medical Information Network Clinical Trials Registry).

Date of registration: From 1 June 2019 to 31 October 2020.

## Introduction

Nocturnal polyuria, which is defined as “excessive production of urine during the individual’s main sleep period”^1^, is considered to be caused by various medical conditions. Nocturnal polyuria is the most common cause of nocturia, accounting for 67%–88% of all nocturia cases^2,3^. Nocturia is defined as “the number of times an individual passes urine during their main sleep period”^1^. Nocturia is a common lower urinary tract symptom in older people, and its incidence is estimated to be > 60% in older people aged > 65 years^4^. Nocturia is not only a troublesome symptom that affects quality of life^5^ but has also recently been shown to be a risk factor for depression and death^6,7^. Therefore, treating nocturia may improve the quality of life in older people and reduce the risk of depression and death. However, the pathogenesis of nocturnal polyuria is complex and not well understood, and no fundamental treatment has been established. This is because research is scarce on clarification of the mechanism of nocturnal polyuria. Many epidemiological studies have reported involvement of various diseases and lifestyles in nocturnal polyuria, but most studies were cross-sectional and did not accurately determine the influence of each factor^8^. To establish a treatment for nocturia, the pathogenesis of nocturnal polyuria should be clarified.

Because many patients with chronic kidney disease (CKD) present with nocturnal polyuria, renal dysfunction is considered to be one of the causes of nocturnal polyuria. A CKD-induced deficit in urinary concentration and increased salt excretion may be the mechanism that leads to nocturnal polyuria^9^. However, no studies have proved a causal relationship between renal dysfunction and nocturnal polyuria or determined its mechanism, and the exact causal relationship and mechanism are not well understood. Therefore, in this study, we aimed to examine the mechanism of nocturnal polyuria in renal dysfunction by prospectively investigating the changes in daytime and nighttime urine volume and urine salt excretion before and after nephrectomy in the same individuals. Identification of the mechanism of nocturnal polyuria is expected to lead to establishment of a treatment strategy for nocturnal polyuria and nocturia.

## Material and method

### Study participants and study design

We included patients who underwent laparoscopic nephrectomy at Osaka University Hospital from June 2019 to October 2020 in this study. The patients were admitted to the hospital 2–4 days before surgery. Blood and urine collection tests were prospectively performed 2 days before and 7 days after nephrectomy to investigate the changes in renal function and daytime and nighttime urine volume, urine osmolality, and salt excretion. Laparoscopic nephrectomy was performed using the retroperitoneal approach and all surgical manipulations were performed extraperitoneally. The day after surgery, intravenous fluid was discontinued and oral food intake was started. Patients with hydronephrosis preoperatively, patients on hemodialysis, and patients with diabetes and positive urine glucose were excluded.

This study was performed in line with the principles of the Declaration of Helsinki. Approval was granted by the Ethics Committee of Osaka University Graduate School of Medicine (No. 18418). Informed consent to participate in the study was obtained from all individual participants included in the study. Informed consent for publication was obtained from all individual participants for whom identifying information is included in this article.

### Evaluation of renal function

Renal function was assessed using the estimated glomerular filtration rate (eGFR). The eGFR was calculated from serum creatinine (sCr) levels in early morning fasting blood samples using the following equation: eGFR (mL/min/1.73 m^2^) = 194 × sCr^−1.094^ × age^−0.287^ × 0.739 (if female)^10^.

### Evaluation of urinary parameters

Twelve-hour urine collection tests were performed, and urine collected from 10:00 to 22:00 h was defined as daytime urine, and urine from 22:00 to 10:00 h as nighttime urine. Urinary sodium (U_Na_), potassium (U_K_), urea nitrogen (U_UN_), and creatinine (U_Cr_) were measured in each period. The nighttime urine volume rate was defined as nighttime urine volume/daily urine volume. Urine osmolality was calculated using the following formula: urine osmolality (mOsm/kg) = 2 × (U_Na_ + U_K_) + U_UN_/2.8. The nighttime salt excretion rate was defined as nighttime salt excretion/daily salt excretion. Fractional excretion of sodium (FE_Na_) was calculated using the following formula: FE_Na_ (%) = (U_Na_/serum Na)/(U_Cr_/sCr) × 100.

### Statistical analysis

Results are expressed as the mean ± SEM or median with interquartile range (IQR). The significance of differences between preoperative and postoperative data was tested using the paired *t*-test. Multiple linear regression analysis was performed to identify independent variables to determine the increase in the nighttime urine volume rate. In this analysis, the preoperative nighttime urine volume rate and the preoperative excretion rate of salt, potassium (K), and urea nitrogen (UN) were covariates. The significance of difference between the change in rate of the eGFR (decrease) and daytime FE_Na_ was tested using the Wilcoxon signed rank test. A *P* value < 0.05 was considered statistically significant. All statistical tests were performed using JMP 14 (SAS Institute, Cary, NC).

## Results

### Participants’ characteristics

There were 49 participants in this study. The characteristics of the participants are shown in Table 1.

**Table 1:** Characteristics of the subjects (n = 49).

Median age, years (range)	64 (39–89)
**No. male (%)**	**24 (49)**
**No. reasons for nephrectomy (%)**	
Kidney transplant donor	36 (73)
Renal cancer	11 (22)
Renal pelvis cancer	2 (4)
Median Operative time, min (Range)	210 (123–374)
Median Blood loss, mL (Range)	20 (0–140)
**No. comorbidities (%)**	
Hypertension	17 (35)
Dyslipidemia	6 (12)
Diabetes mellitus	3 (6)
Cerebrovascular disease	2 (4)
Malignant tumors	2 (4)

### Changes in the eGFR

**Figure 1:**
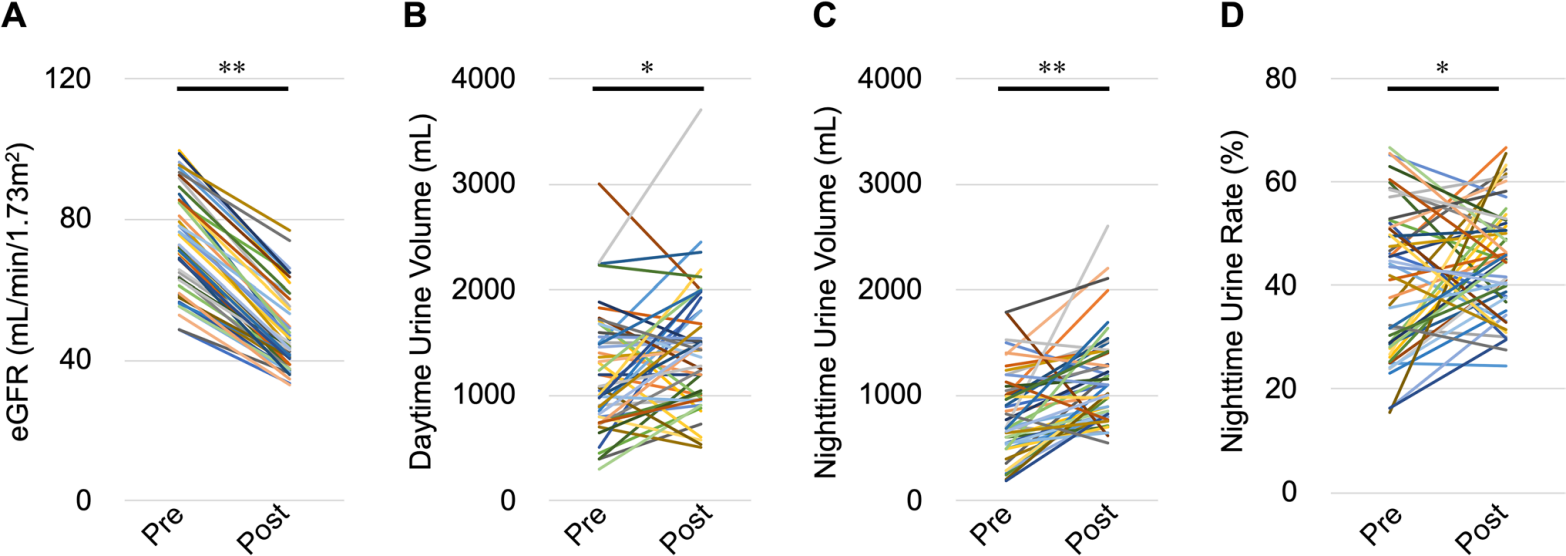
Changes in renal function and urine volume caused by nephrectomy. (**A**) The eGFR, (**B**) daytime urine volume, (**C**) nighttime urine volume, and (**D**) the nighttime urine volume rate. *eGFR* estimated glomerular filtration rate, *Pre* preoperative, *Post* postoperative. *P < 0.05, **P < 0.01, by the paired *t*-test.

The eGFR was significantly decreased after nephrectomy (47.2 ± 1.6 mL/min/1.73 m^2^) compared with before nephrectomy (73.3 ± 2.0 mL/min/1.73 m^2^, *P* < 0.01) (Fig. 1A).

### Changes in daytime and nighttime urine volume

Nephrectomy significantly increased daytime urine volume (from 1214 ± 79 to 1431 ± 84 mL, *P* = 0.01) and nighttime urine volume (from 820 ± 57 to 1155 ± 62 mL, *P* < 0.01) (Fig. 1B,C), and increased the nighttime urine volume rate (from 40.6% ± 2.0% to 45.3% ± 1.5%, *P* = 0.04) compared with before nephrectomy (Fig. 1D). These findings indicate that decreased renal function increases the nighttime urine volume rate.

### Changes in daytime and nighttime urine osmolality and excretion of salt, K, and UN

**Figure 2:**
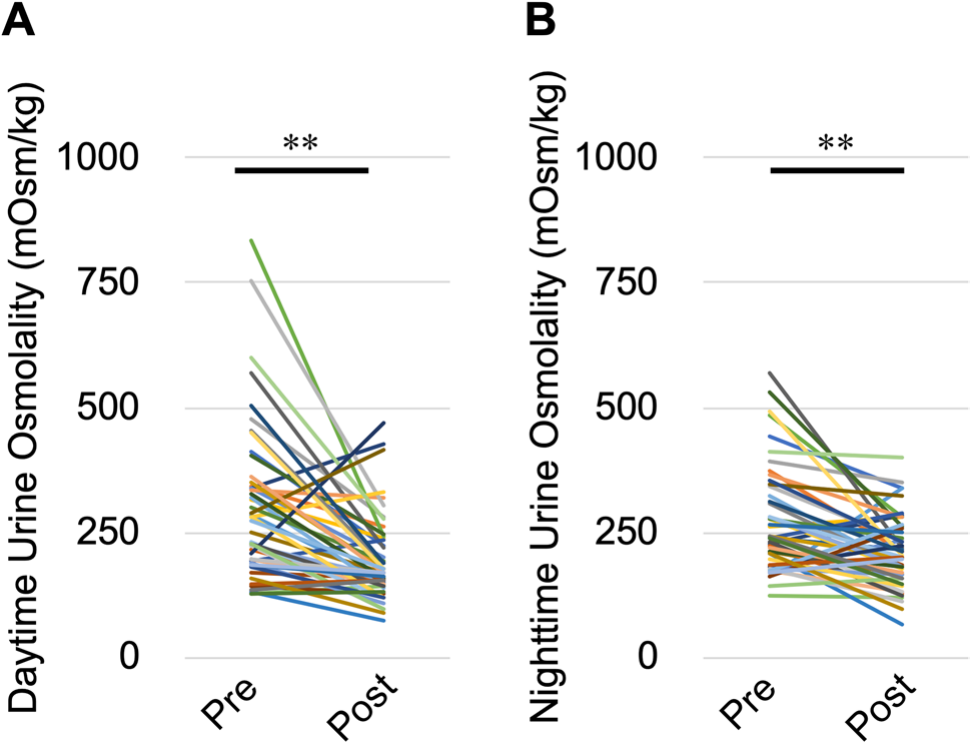
Changes in urine osmolality caused by nephrectomy. (**A**) Daytime and (**B**) nighttime urine osmolality. *Pre* preoperative, *Post* postoperative. **P < 0.01, by the paired *t*-test.

**Figure 3:**
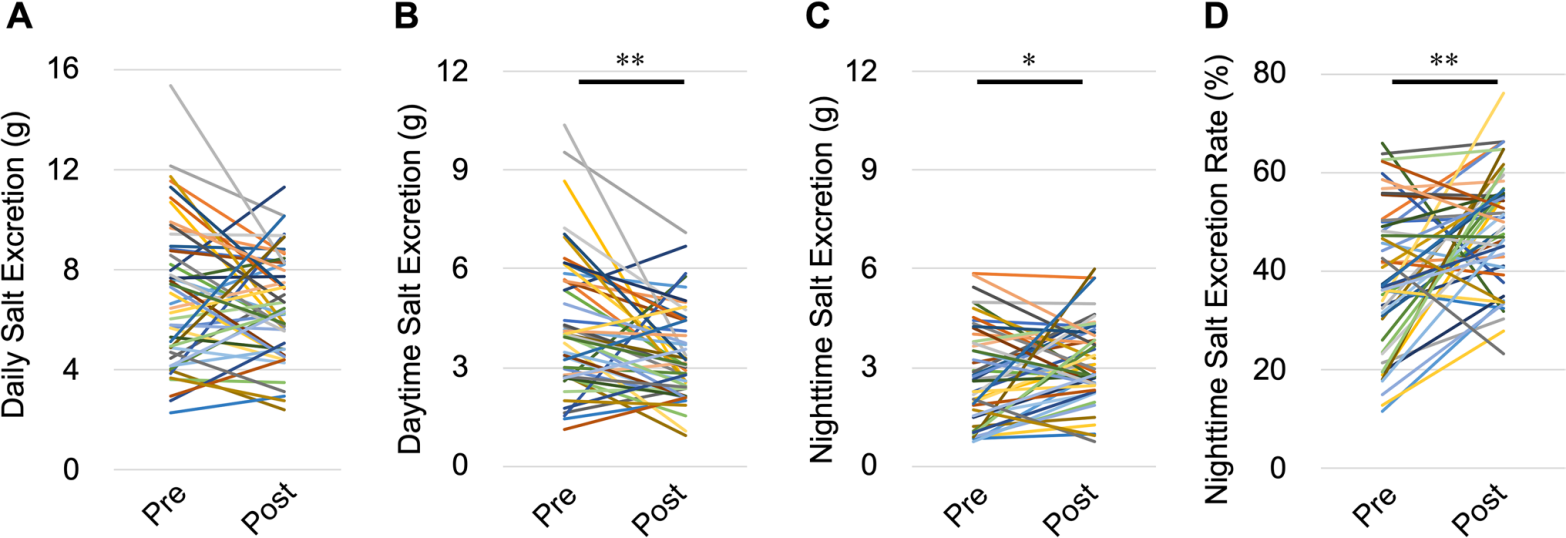
Changes in salt excretion caused by nephrectomy. (**A**) Daily salt excretion, (**B**) daytime salt excretion, (**C**) nighttime salt excretion, and (**D**) the nighttime salt excretion rate. *Pre* preoperative, *Post* postoperative. *P < 0.05, **P < 0.01, by the paired *t*-test.

**Figure 4:**
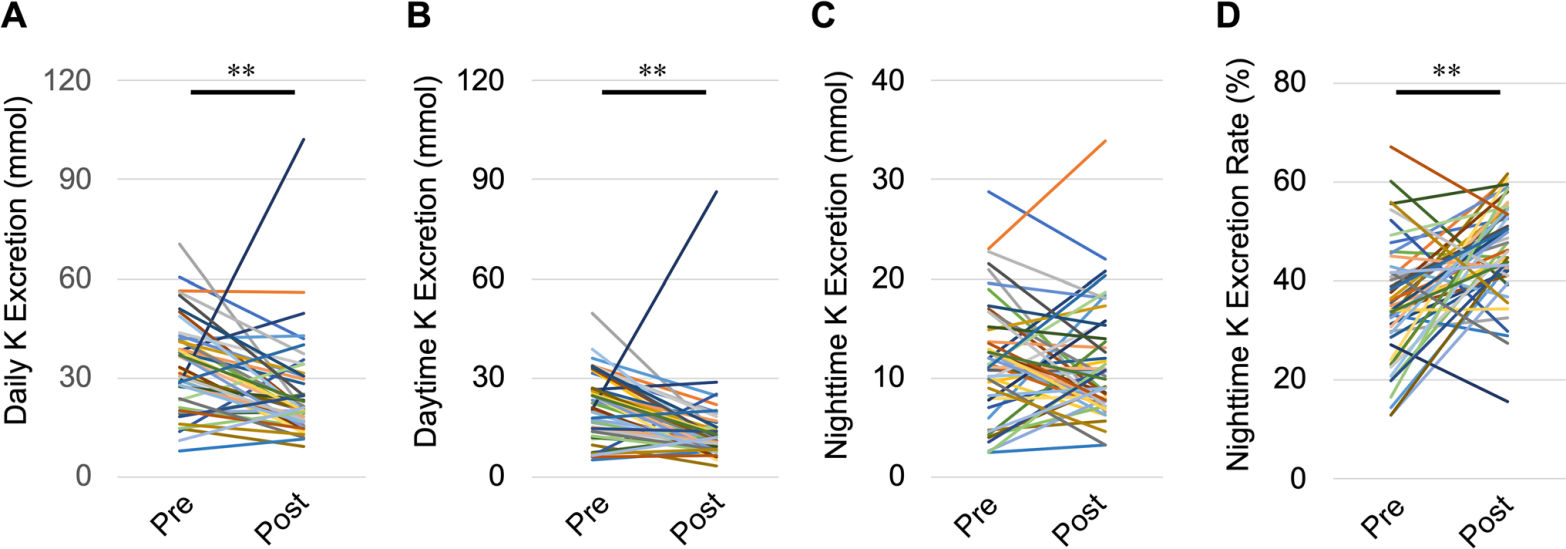
Changes in K excretion caused by nephrectomy. (**A**) Daily K excretion, (**B**) daytime K excretion, (**C**) nighttime K excretion, and (**D**) the nighttime K excretion rate. *Pre* preoperative, *Post* postoperative, *K* potassium. **P < 0.01, by the paired *t*-test.

**Figure 5:**
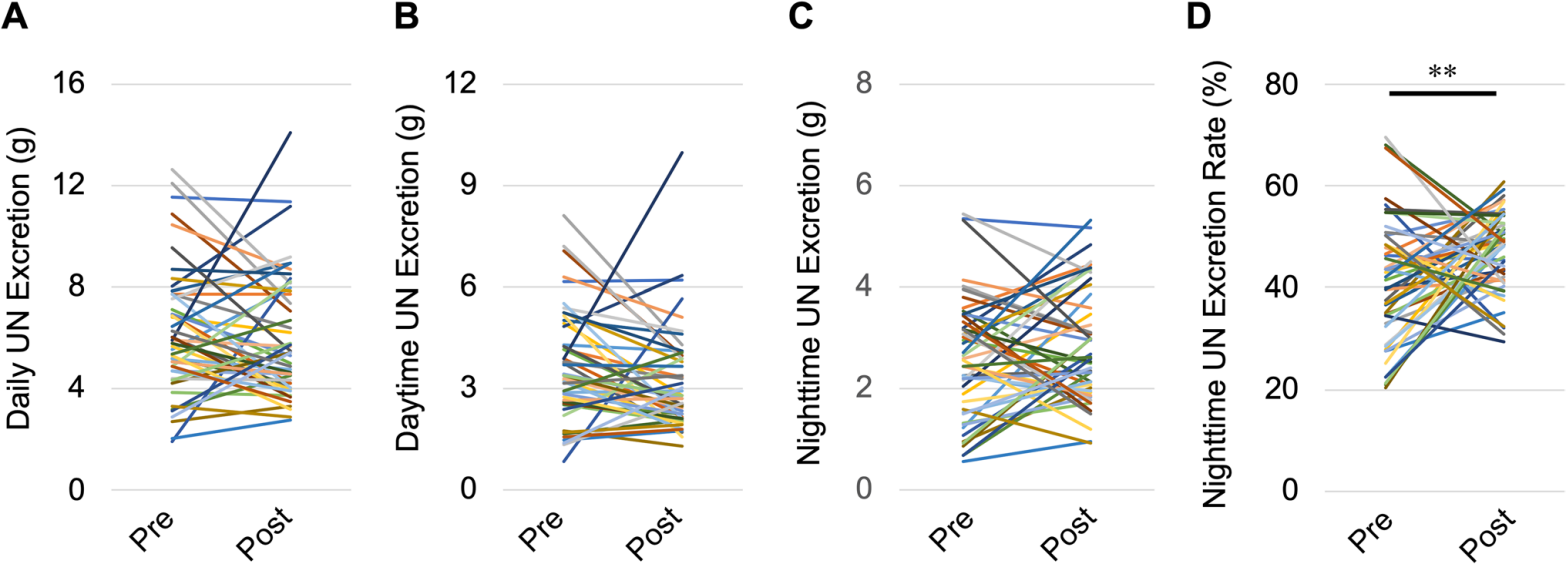
Changes in UN excretion caused by nephrectomy. (**A**) Daily UN excretion, (**B**) daytime UN excretion, (**C**) nighttime UN excretion, and (**D**) the nighttime UN excretion rate. *Pre* preoperative, *Post* postoperative, *UN* urea nitrogen. **P < 0.01, by the paired *t*-test.

Nephrectomy significantly decreased daytime urine osmolality (from 300 ± 22 to 195 ± 12 mOsm/kg, *P* < 0.01) and nighttime urine osmolality (from 273 ± 15 to 212 ± 10 mOsm/kg, *P* < 0.01) compared with before nephrectomy (Fig. 2A,B). There was no significant difference in daily salt excretion between before and after nephrectomy (7.07 ± 0.41 vs. 6.55 ± 0.30 g, *P* = 0.1723). However, nephrectomy significantly decreased daytime salt excretion (from 4.33 ± 0.29 to 3.36 ± 0.20 g, *P* < 0.01), increased nighttime salt excretion (from 2.74 ± 0.21 to 3.20 ± 0.17 g, *P* = 0.04), and increased the nighttime salt excretion rate (from 38.7% ± 2.1% to 48.8% ± 1.7%, *P* < 0.01) compared with before nephrectomy (Fig. 3A–D). Nephrectomy also significantly decreased daily K excretion (from 33.4 ± 2.0 to 25.4 ± 2.2 mEq, *P* < 0.01) and daytime K excretion (from 21.7 ± 1.4 to 14.0 ± 1.7 mEq, *P* < 0.01) and increased the nighttime K excretion rate (from 35.5% ± 1.7% to 46.5% ± 1.4%, *P* < 0.01) compared with before nephrectomy. There was no significant difference in nighttime K excretion after nephrectomy compared with before nephrectomy (11.7 ± 0.8 vs. 11.4 ± 0.8 mEq, *P* = 0.750) (Fig. 4A–D). Nephrectomy significantly increased the nighttime UN excretion rate compared with before nephrectomy (from 41.3% ± 1.8% to 47.4% ± 1.1%, *P* < 0.01). However, there were no significant differences in daily UN excretion (6.23 ± 0.36 vs. 6.01 ± 0.35 g, *P* = 0.559), daytime UN excretion (3.65 ± 0.23 vs. 3.19 ± 0.22 g, *P* = 0.074) and nighttime UN excretion (2.57 ± 0.17 vs. 2.83 ± 0.16 g, *P* = 0.174) after nephrectomy compared with before nephrectomy (Fig. 5A–D). Although K excretion was significantly decreased, there was no significant difference in the total amount of urine osmolytes (i.e., 2Na^+^, 2 K^+^, and urea osmolyte) (530.8 ± 28.8 vs. 490.0 ± 24.5 mmol, *P* = 0.28). These findings indicate that decreased renal function causes carryover of salt, K, and UN excretion to the nighttime.

### Factors associated with the increase in the nighttime urine volume rate in renal dysfunction

The results mentioned above indicate that impaired renal function decreases urine osmolality and increases the nighttime excretion rate of salt, K, and UN. All of these changes could affect the nighttime urine volume rate. To identify the factors associated with an increased nighttime urine volume rate with impaired renal function, we conducted multiple linear regression analysis. This analysis showed that age, a change in nighttime urine osmolality, and a change in the nighttime salt excretion rate were significantly associated with the nighttime urine volume rate (all *P* < 0.01). Of these factors, a change in the nighttime salt excretion rate had the highest standard partial regression coefficient and had the strongest effect on the nighttime urine volume rate (Table 2).

**Table 2:** Factors associated with the increase in the nighttime urine volume rate.

Variables	Simple linear regression			Multiple linear regression			
	Coefficient	(95% CI)	*P*	Unstandardized coefficient	(95% CI)	Standardized coefficient	*P*
Age	0.10	(− 0.27, 0.48)	0.59	0.27	(0.12, 0.42)	0.21	< 0.01
Pre eGFR	− 0.01	(− 0.35, 0.32)	0.94				
ΔeGFR	− 0.13	(− 0.82, 0.55)	0.70				
ΔNighttime urine osmolality	− 0.02	(− 0.07, 0.02)	0.35	0.03	(0.01, 0.05)	0.17	< 0.01
ΔNighttime salt excretion rate	0.73	(0.55, 0.91)	< 0.01	0.75	(0.48, 1.03)	0.79	< 0.01
ΔNighttime K excretion rate	0.69	(0.48, 0.91)	< 0.01	− 0.36	(− 0.73, 0.01)	− 0.36	0.06
ΔNighttime urea excretion rate	0.79	(0.60, 0.99)	< 0.01	0.42	(− 0.02, 0.86)	0.40	0.06

*Pre* Preoperative, *eGFR* estimated glomerular filtration rate.

### Changes in daytime and nighttime FE_Na_

**Figure 6:**
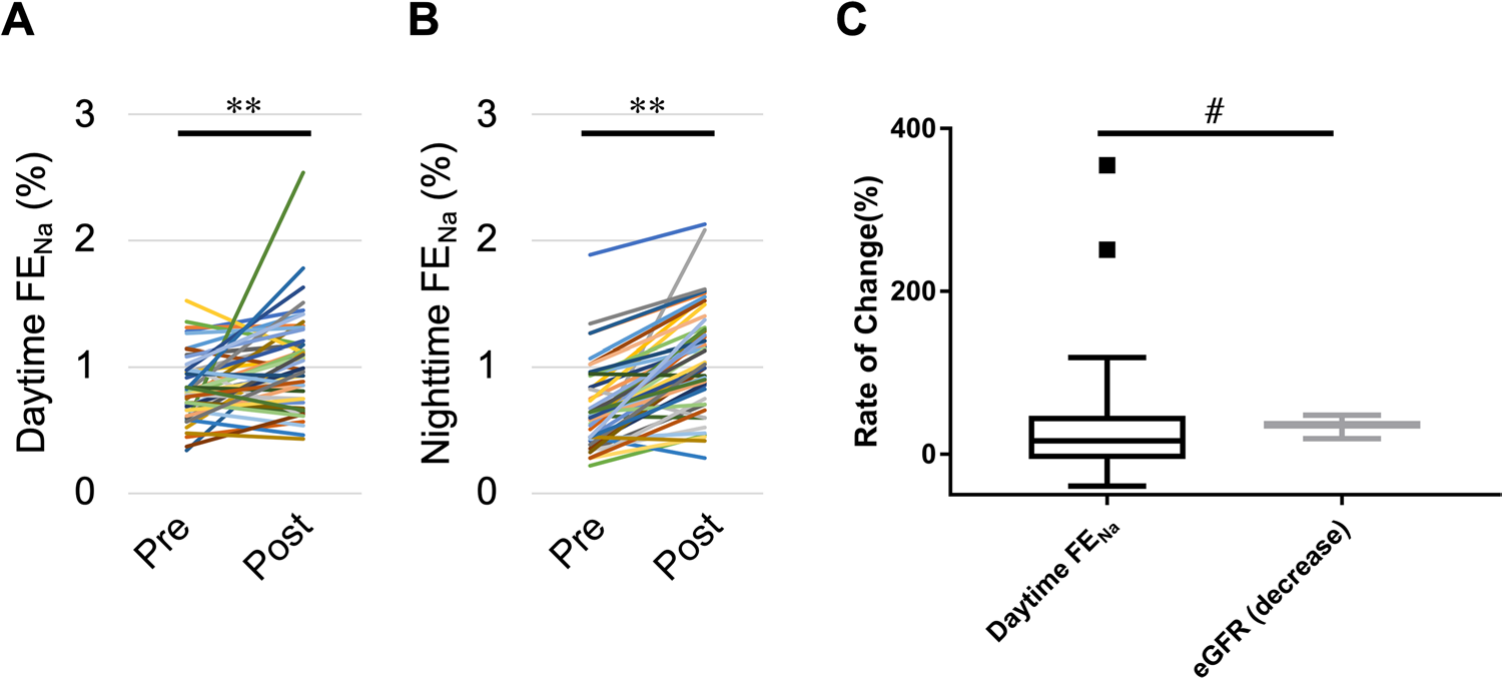
Changes in FE_Na_ and the GFR caused by nephrectomy. (**A**) Daytime FE_Na_ and (**B**) nighttime FE_Na_ in each patient. (**C**) Tukey’s box and whisker plots for the rate of change in daytime FE_Na_ and the eGFR (decrease). *FE*_*Na*_ fractional excretion of sodium; *eGFR* estimated glomerular filtration rate, *Pre* preoperative, *Post* postoperative. **P < 0.01, by the paired *t*-test; #*P* < 0.05, by the Wilcoxon signed rank test.

To investigate the causes of the increase in the nighttime salt excretion rate (i.e., carryover of salt excretion to the nighttime after nephrectomy) we compared changes in the eGFR and FE_Na_ with nephrectomy. Nephrectomy significantly increased daytime FE_Na_ (from 0.83% ± 0.04% to 1.03% ± 0.05%, *P* < 0.01) and nighttime FE_Na_ (from 0.66% ± 0.05% to 1.07% ± 0.06%, *P* < 0.01) compared with before nephrectomy (Fig. 6A,B). The rate of change (increase) in daytime FE_Na_ (16.7%; IQR, − 5.8 to 47.3) was significantly lower than the rate of change (decrease) in the eGFR (35.9%; IQR, 31.48 to 41.05, *P* = 0.03) (Fig. 6C).

## Discussion

In this study, we prospectively investigated the changes in urine volume, urine osmolality, and urinary excretion of salt, K, and UN during the daytime and nighttime before and after nephrectomy. We found that the nighttime urine volume rate increased as renal function decreased. We also found an increase in the nighttime salt, K, and UN excretion rates. The mechanism of urine concentrations is mainly regulated by three major urinary osmolytes, Na, K, and Urea^11,12^. Therefore, in our study, multiple linear regression analysis was performed to identify independent variables to determine the increase in the nighttime urine volume rate. This analysis showed that the increase in nighttime salt excretion was most strongly related to the increase in the nighttime urine volume rate with decreased renal function. After nephrectomy, despite the decrease in daily salt excretion, nighttime salt excretion increased, which suggests that renal dysfunction causes an increase in nighttime salt excretion. Furthermore, we found that the rate of change (increase) in daytime FE_Na_ was significantly lower than the rate of change (decrease) in the eGFR. This insufficient increase in daytime FE_Na_ compared with a decrease in the eGFR may be the cause of the decrease in daytime salt excretion. This finding suggests that renal dysfunction decreases daytime salt excretion, which results in increased salt excretion at nighttime. Our findings suggest that the main cause of the increase in the nighttime urine volume rate in renal dysfunction is carryover of salt excretion to the nighttime.

The reason why FE_Na_ did not increase as the eGFR decreased in our study is unclear. A decrease in the glomerular filtration rate (GFR) results in an increase in FE_Na_, which maintains Na balance. An example of this situation is when the GFR decreases by half, FE_Na_ increases by two fold^13^. This antagonism between a decrease in the GFR and an increase in FE_Na_ is thought to be an essential mechanism for the body to maintain Na balance. However, the changes between daytime and nighttime have not been well investigated. In this study, we examined the changes in daytime and nighttime FE_Na_ and found that the increase in daytime FE_Na_ was milder than the decrease in GFR. To the best of our knowledge, there have been no reports that evaluated changes in FE_Na_ separately for daytime and nighttime. Although we do not know the exact mechanism of our finding, there might not be a system present in the human body for rapid excretion of excess salt intake. In the process of evolution, humans have developed a urine reabsorption system in the kidney, including the glomerulus, tubule, and Henle’s loop, and can reabsorb more than 99% of Na^14^. People can survive on a small amount of salt, as shown by the Yanomamo, who survive on a salt intake of < 1 g/day^15^. However, most people consume approximately 10 g of salt a day, which is believed to be too high. Various diseases in the current era, such as hypertension and stroke, are related to excessive salt intake^16^. Humans have only been consuming this high amount of salt for approximately 5,000–10,000 years^17^ and have therefore not yet acquired the ability to rapidly excrete excess salt.

Recently, nocturia, which is a common lower urinary tract symptom in older people, has been found to be not only a cause of decreased quality of life, but also a risk factor for depression and death^6,7^. The causes of nocturia include global polyuria, nocturnal polyuria, bladder storage problems, and sleep disorders^18^, of which the most common cause is nocturnal polyuria^2^. Nocturnal polyuria may be a multifactorial condition with many possible contributing factors, including behavioral, physiological, and pathological factors. Renal dysfunction is considered to be one of the causes of nocturnal polyuria because nocturnal polyuria is one of the initial symptoms in patients with CKD. A decrease in urine concentrations and an increase in nocturnal salt excretion may be a cause of nocturnal polyuria in decreased renal function^9^. However, previous studies on this issue were cross-sectional and had limited ability to prove causality. In the present study, we performed prospective analysis of changes in urine volume, urine osmolality, and salt excretion during the daytime and nighttime before and after nephrectomy. We found that the increase in nocturnal urine volume associated with renal dysfunction was due to carryover of salt excretion to the nighttime.

Excessive salt intake has attracted attention as one of the causes of nocturnal polyuria^19,20^. However, there have also been negative reports on the relationship between nocturnal polyuria and excessive salt intake^21,22^. Therefore, whether excessive salt intake is a cause of nocturnal polyuria remains controversial. In this study, we investigated the effect of decreased renal function on nocturnal polyuria and found that salt excretion was carried over to the nighttime and the nocturnal urine volume rate increased when renal function decreased. This suggests that renal dysfunction is involved in the association between excessive salt intake and nocturnal polyuria. Therefore, when people with impaired renal function consume excessive salt, salt excretion may be carried over to the nighttime, resulting in nocturnal polyuria. In cases of nocturia due to this mechanism, a reduction in salt intake or administration of diuretics, such as thiazide, may be useful in reducing nocturnal salt excretion and nocturnal urine. However, the criteria for excessive salt intake in terms of nocturnal polyuria are unknown, and future research is required to establish the criteria of optimal salt intake.

There are some limitations to this study. First, this was a one-arm study and we did not have controls. Therefore, we were not able to accurately assess the effect of decreased renal function. Surgical stress may cause a variety of stress responses to the patient, which may affect urine production and salt excretion. An example of this stress response is that cortisol, which a stress hormone, causes retention of Na and may affect salt excretion. A study of patients undergoing non-nephrectomy surgery as controls would have allowed us to accurately assess the effect of decreased renal function on nocturnal polyuria. However, in this study, the level of surgical stress was considered to be moderate because all surgeries were performed laparoscopically and extraperitoneally, with a short operative time, minimal blood loss, and minimal intravenous fluid infusion. In fact, a previous study showed that, in patients who underwent laparoscopic nephrectomy, blood cortisol levels increased immediately after surgery, but returned to preoperative levels 1 day after surgery^23^. Therefore, we believe that the effect of the stress response on the conclusion of this study may be limited. Second, because we defined urine collected from 22:00 to 10:00 h as nighttime urine, nighttime urine could have contained salt from breakfast, which may have affected the results. We decided to use this definition for two reasons. The first reason was to ensure that salt excreted during the nighttime was evaluated as nighttime salt excretion. Because the main purpose of this study was to evaluate nephrectomy-induced changes in urine volume and salt excretion between daytime and nighttime, the first morning urine needed to be evaluated as nighttime urine. The timing of the first morning urine varies in each patient. Some patients void immediately after waking up, while others void after breakfast. The second reason is that the effect of breakfast on urine obtained from 22:00 to 10:00 h is considered to be decreased after nephrectomy. Salt excretion is delayed as renal function decreases^13^. Therefore, the nephrectomy-related increase in nighttime salt excretion is not considered to be associated with salt intake at breakfast but associated with increased salt excretion during the nighttime. For these two reasons, we believe that the definition of nighttime urine in this study does not affect our conclusions.

## Conclusions

The present study shows that a decrease in renal function causes a decrease in nighttime urine osmolality and an increase in the nighttime salt, K, and UN excretion rates. Among these factors, the increase in the nighttime salt excretion rate was found to have the strongest effect on the increase in the nighttime urine volume rate. These results suggest that decreased renal function increases the nighttime urine volume rate by carryover of salt excretion to the nighttime.

## Data Availability

All data generated or analyzed during this study are included in this published article.
